# Flexible, transparent patterned electrodes based on graphene oxide/silver nanowire nanocomposites fabricated utilizing an accelerated ultraviolet/ozone process to control silver nanowire degradation

**DOI:** 10.1038/s41598-019-41909-4

**Published:** 2019-04-02

**Authors:** Dong Chul Choo, Sang Kyung Bae, Tae Whan Kim

**Affiliations:** 1 Department of Electronics and Computer Engineering, Hanyang University, Seoul, 04763 Republic of Korea; 2 Department of Information Display Engineering, Hanyang University, Seoul, 04763 Republic of Korea

## Abstract

We developed flexible, transparent patterned electrodes, which were fabricated utilizing accelerated ultraviolet/ozone (UV/O_3_)-treated graphene oxide (GO)/silver nanowire (Ag-NW) nanocomposites via a simple, low-cost pattern process to investigate the feasibility of promising applications in flexible/wearable electronic and optoelectronic devices. The UV/O_3_ process of the GO/Ag-NW electrode was accelerated by the pre-heat treatment, and the degradation interruption of Ag NWs was removed by the GO treatment. After the deposition of the GO-treated Ag NW electrodes, the sheet resistance of the thermally annealed GO-treated Ag-NW electrodes was significantly increased by using the UV/O_3_ treatment, resulting in a deterioration of the GO-treated Ag NWs in areas exposed to the UV/O_3_ treatment. The degradation of the Ag NWs caused by the UV/O_3_ treatment was confirmed by using the sheet resistances, scanning electron microscopy images, X-ray photoelectron microscopy spectra, and transmittance spectra. While the sheet resistance of the low-density Ag-NW electrode was considerably increased due to the pre-thermal treatment at 90 °C for 10 min, that of the high-density Ag-NW electrode did not vary significantly even after a UV/O_3_ treatment for a long time. The degradation interference phenomenon caused by the UV/O_3_ treatment in the high-density Ag NWs could be removed by using a GO treatment, which resulted in the formation of a Ag-NW electrode pattern suitable for promising applications in flexible organic light-emitting devices. The GO treatment decreased the sheet resistance of the Ag-NW electrode and enabled the pattern to be formed by using the UV/O_3_ treatment. The selective degradation of Ag NWs due to UV/O_3_ treatment decreased the transparency of the Ag-NW electrode by about 8% and significantly increased its sheet resistance more than 100 times.

## Introduction

Flexible electronic devices have been attractive because of interest in potential applications in next-generation intelligent systems^1–3^. Rapid advances in the development of silver-nanowire (Ag-NW) electrodes have made possible flexible, transparent electrodes^4–6^. Light-emitting devices fabricated utilizing flexible, transparent substrates have the possibility of being applied to various designs requiring strong impact resistance and high portability^7–9^. Among the various flexible, transparent electrodes, Ag-NW, graphene, and metal-mesh electrodes have emerged as excellent candidates for low-cost flexible, transparent electrodes^9–13^. Furthermore, the solution process used to fabricate the Ag-NW electrodes is simple and inexpensive to use, and the transmittances and the sheet resistances of the resulting Ag-NW electrodes are superior to those of other flexible electrodes. However, the commercialization of transparent Ag-NW electrodes has been hindered by their inherent problems: image blurring due to the haze phenomenon, very large surface roughness, and difficult pattern-formation process^14–16^. Due to improvements in the synthesis process, Ag NWs can be made thinner and longer, which would dramatically decrease the haze phenomenon associated with Ag-NW electrodes. Because the haze associated with Ag-NW electrodes can be reduced to less than 3% due to such improvements, the performances of those electrodes will not be significantly degraded compared with the performances of conventional transparent electrodes^17^. Moreover, the large surface roughness of Ag-NW electrodes can be reduced to a few nanometers by synthesizing ultra-thin Ag NWs, applying a planarization layer with a sufficient thickness, or transferring the Ag NWs to a polymer substrate^18–20^.

A laser-patterning method has been extensively used to form Ag-NW patterns on electrodes^21^. Even though this method can form fine, high-quality Ag-NW patterns, because the process cost is very high, using this method to produce a large-sized product is difficult. Various patterning methods, such as the transfer of the Ag-NW patterns to a polymer substrate, photolithography, direct patterning, plasma etching, and magnetic printing, have been proposed as ways to overcome this inherent problem^22–28^. However, the methods used to form Ag-NW patterns involve complicated solutions and high-temperature processes, and they are still too complicated to be applied commercially to the fabrication of large-sized products.

This paper reports a novel method for forming Ag-NW patterns on electrodes by using a thermal treatment, a graphene-oxide (GO) layer, and an ultraviolet/ozone (UV/O_3_) treatment. The GO-treated Ag-NW electrode was patterned by using a thermal annealing and a UV/O_3_ treatment through a mask in an atmospheric condition, which can be easily used in the production of large-area devices. Even though the thermal and UV/O_3_ treatments are relatively very simple processes, the combination of the two processes can effectively form a large-area Ag-NW electrode pattern. While the degradation of the GO-treated Ag-NW electrode due to the UV/O_3_ treatment was actually very slow, the pre-thermal treatment was able to decrease the degradation time effectively by changing the surface composition of the Ag-NWs. This pattern formation method can be combine with the various coating methods, resulting in a formation of more effective and lower cost process. While the conventional laser pattern method requires a high cost for enlarging the pattern area, it is possible to form a large area pattern at low cost by adding UV lamps necessary for the UV/O_3_ process used in this method. Furthermore, the patterning method proposed in this manuscript requires the same processing time even when the substrate area increases. The sheet resistances and the transmittance spectra were measured to investigate the electrical and the optical properties of the Ag-NW-patterned electrodes. Scanning electron microscopy (SEM) and X-ray photoelectron spectroscopy (XPS) measurements were performed to investigate the structural properties and the chemical compositions of those electrodes.

## Methods

### Sample preparation

Polyethylene terephthalate (PET) substrates were cleaned with methanol for 10 min, after which they were thoroughly rinsed in de-ionized water. After the PET substrates had been dried by using N_2_ gas with a purity of 99.99%, the surfaces of the substrates were treated with a UV/O_3_ treatment for 20 min. Low-density Ag-NW electrodes were fabricated by spin coating a Ag-NW solution (purchased from Nanopyxis Co.) onto the cleaned PET substrates at 6000 rpm for 30 s and then drying them in the atmosphere for approximately 1 h. The sheet resistance of the low-density Ag-NW electrode decreased from 280 to 250 Ω/sq as the result of a thermal treatment at 90 °C for 10 min on a hot plate. A GO treatment was performed by spin coating a solution of GO flakes dispersed in isopropyl alcohol at 2000 rpm for 30 s. After the GO treatment, the Ag-NW electrode was dried in air for 1 h and then thermally treated again at 90 °C for 10 min by using a hot plate.

The high-density Ag NW electrodes were fabricated by using a bar-coating method. After an A4-sized PET sheet was wiped with methanol, the PET sheet was rinsed with deionized water. After the PET sheet was dried with N_2_ gas, the PET sheet was treated with a UV/O_3_ treatment for 20 min. A diluted Ag-NW solution was bar-coated twice onto the cleaned PET sheet with a Meyer bar #10. The Ag-NW-coated PET sheet was dried in air for 1 h, after which it was cut into 2.5 × 2.5 cm^2^ pieces. Those pieces were further processed by using a GO treatment with a spin-coater or a thermal treatment on a hot plate. The UV/O_3_ treatment using a UV/ozone cleaner exposed the Ag-NW electrodes to UV radiation at wavelengths from 185 to 254 nm.

### Electrical, optical, chemical, and structural measurements

The sheet resistances were measured by using a sheet resistance meter (FPP-40K, DASOL ENG) and were obtained by measuring nine points on the substrate and determining the mean value and the standard deviation. The transmittance spectra were measured by using a UV-visible spectrometer (Lambda 650 S, Perkin Elmer). The structural properties were investigated by using SEM (NOVA NANOSEM 450, FEI) while the surface chemical compositions were measured by using XPS (XPS-Theta Probe, Thermo Fisher Scientific Co.).

## Results

**Figure 1:**
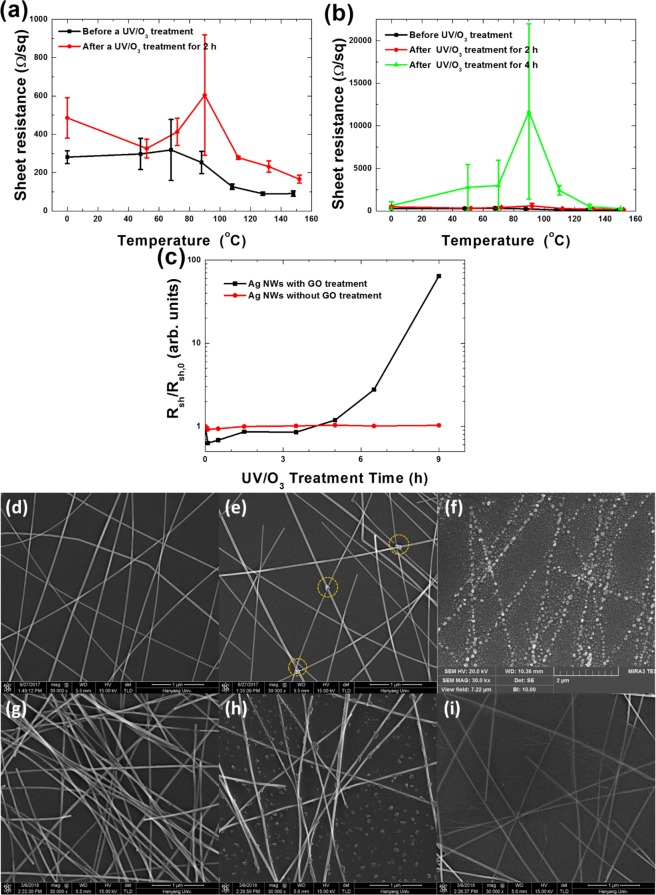
Sheet resistances of the spin-coated Ag NWs with increasing pre-annealing treatment temperature (**a**) before and after UV/O_3_ treatment for 2 h and (**b**) after UV/O_3_ treatment for 4 h, (**c**) sheet resistance ratios as functions of the UV/O_3_ treatment times for the Ag NWs with and without GO treatment. Scanning electron microscopy images of (**d**) Ag NWs spin-coated on PET substrates, (**e**) Ag NWs thermally annealed at 90 °C for 10 min, (**f**) Ag NWs UV/O_3_ treated for 9 h, (**g**) Ag NWs bar coated on a PET substrate, (**h**) Ag NWs UV/O_3_-treated for 9 h, and (i) GO-treated Ag NWs. The yellow dotted circles in (**e**) represents the disconnected regions of the Ag NWs after thermal annealing.

Figure 1 shows the sheet resistances of the spin-coated Ag NWs as functions of the pre-annealing temperature (a) before and after UV/O_3_ treatment for 2 h and (b) before and after UV/O_3_ treatment for 4 h. The sheet resistance of the Ag NWs decreased with increasing thermal treatment temperature, as shown in Fig. 1(a). The sheet resistance of the Ag NWs with the UV/O_3_ treatment decreased rapidly with increasing thermal treatment temperature above 70 °C, which might be related to a decrease in the contact resistance caused by the melting of the Ag NWs at their points of contact. However, when the UV/O_3_ treatment was applied, the sheet resistance was higher at all pre-annealing temperatures, as shown in Fig. 1(a), but the increase in the sheet resistance was largest for the Ag NWs pre-annealed at 90 °C. When the UV/O_3_ treatment was performed on the Ag NWs for a longer time (4 h, rather than 2 h), the sheet resistance of the Ag NW electrodes dramatically increased, as shown in Fig. 1(b). Again, the sheet resistance of the Ag NWs pre-annealed at 90 °C showed the largest increase after UV/O_3_ treatment for 4 h. The deterioration of the Ag-NW electrode due to the UV/O_3_ treatment was dramatically affected by the pre-thermal treatment temperature. While XPS measurements after 10 min of thermal treatment did not detect a valid change in the Ag 3d and the N 1 s spectra, a subtle change was observed in the C1s spectra. When the C 1s spectrum was decomposed into C-C, C-O and C=O peaks, the C-C peak was slightly decreased and the C-O peak was slightly increased as the annealing temperature of the Ag-NW electrode was increased, as shown in Fig. S1. In addition, the C=O peak appeared, and the C-O peak was decreased after thermal treatment at a higher temperature than the optimal temperature. After the UV/O_3_ treatment, the C-O peak was decreased, and the C=O peak was increased. Especially, the C=O peak in the pre-thermally-treated Ag-NW electrode was increased by more than 50% in comparison with that in the as-grown Ag-NW electrode. Table S1 summarizes the percent contributions of the C-C, the C-O, and the C=O peaks to the C1s spectrum of the Ag-NW electrode treated with heat and UV/O_3_. When the thermal treatment temperature was lower than the optimum temperature, the C=O peak did not appear during the pre-thermal treatment, and the increase in the C=O peak due to the UV/O_3_ treatment was significantly enhanced when the UV/O_3_ treatment was performed under the condition that the contribution of the C-O peak was maximum. A transition from the C-C state to the C=O state was caused by the UV/O_3_ treatment, and the pre-thermal treatment increased the contribution of the C-O state with a binding energy intermediate to those of the two states. The contribution of the C-O state was increased by the pre-thermal treatment, and the transition from the C-C state to the C=O state due to the UV/O3 treatment was accelerated by that treatment, resulting in a decrease in the UV/O_3_ degradation time. When the UV/O_3_ treatment on the Ag NWs was carried out for longer than 7 h (data not shown), the sheet resistance increased to more than 100 kΩ/sq. In Fig. 1(c), the filled circles represent the sheet resistance as a function of the UV/O_3_ treatment time for the bar-coated Ag NWs without GO treatment. The surface resistance of the bar-coated Ag NWs did not change significantly with increasing UV/O_3_ treatment time up to 9 h. Even when the UV/O_3_ process time was increased to 24 h, the sheet resistance of the bar-coated Ag NWs did not increase significantly. The surface of the Ag NWs was degraded due to the UV/O_3_ treatment, and the debris from reactions between the ozone and the Ag NWs appeared around the Ag NWs, as shown in Fig. 1(f). Much debris was attached on the surfaces of the Ag NWs, which disturbed the degradation process of the Ag NWs. However, when the UV/O_3_ treatment was applied to the bar-coated Ag NWs covered with GO flakes, their sheet resistance increased after 3 h and increased to more than 60 kΩ/sq after 9 h, as shown by the filled rectangles in Fig. 1(c). Because the debris was formed due to the reaction of ozone at the surfaces of the Ag NWs in the GO-treated Ag NWs, the debris did not interfere with the degradation of nearby Ag NWs, resulting in an increase in the sheet resistance.

Figure 1(d–i) show SEM images of Ag NWs after various treatments. Figure 1(d) shows an SEM image of pristine, spin-coated Ag NWs that had undergone neither thermal nor UV/O_3_ treatment. Figure 1(e) presents an SEM image of the Ag NWs thermally annealed at 90 °C for 10 min. Shape variations can be seen at the points where the thermally annealed Ag NWs come into contact with one another. These shape variations might reduce the contact resistance between the Ag NWs^29^. However, when the annealing temperature was increased, the sheet resistance of the Ag NWs increased due to the Ag NWs becoming disconnected. The disconnections of the Ag NWs were denoted by the yellow dotted circles, as shown in Fig. 1(e). Figure 1(f) shows that the Ag NWs were completely destroyed after a UV/O_3_ treatment for 9 h. Ozone formed by UV irradiation reacted with the polyvinylpyrrolidone (PVP) protective layer surrounding the Ag NWs or with the Ag NW surface, resulting in the temporary formation of silver nitride or silver oxide. The silver nitride or silver oxide rapidly decomposed and changed the surface morphology of the Ag NWs, resulting in the destruction of the Ag-NW network after a sufficient reaction time. However, when the Ag NW electrodes were coated by using a bar-coating method, the density of the Ag NWs was increased, as shown in Fig. 1(g), and their sheet resistance decreased to approximately 50 Ω/sq. While the sheet resistance of the spin-coated Ag NWs was larger after UV/O_3_ treatment, that of the bar-coated Ag NWs was not. The SEM images for the bar-coated Ag NWs on a PET substrate before and after a UV/O_3_ treatment for 10 h are shown in Fig. 1(g,h), respectively. When the density of the Ag NWs was high, the sheet resistance did not increase because the Ag-NW network was not broken even when the UV/O_3_ treatment was continued for a long time. The degradation of the Ag NWs was confirmed by analyzing the variations in the transmittance spectra.

**Figure 2:**
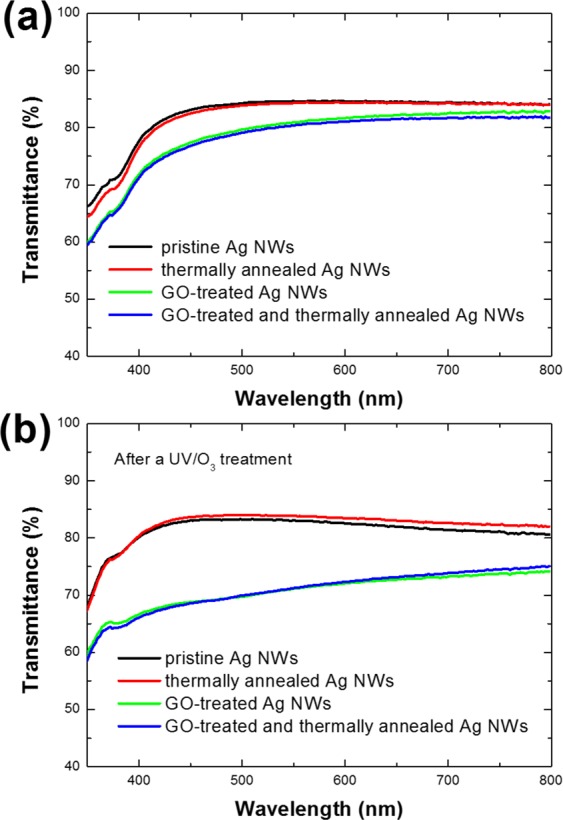
Transmittance spectra of pristine, thermally-annealed, GO-treated, and thermal-annealed Ag-NW electrodes after a GO treatment (**a**) before a UV/O_3_ treatment and (**b**) after a UV/O_3_ treatment for 9 h.

Figure 2 shows the transmittance spectra of pristine, thermally annealed, GO-treated, and thermally annealed, GO-treated Ag NWs (a) before UV/O_3_ treatment and (b) after UV/O_3_ treatment for 9 h. In Fig. 2(a), while the average transmittance at the wavelengths of visible light between 380 and 780 nm was 83.2%, their transmittances after the thermal annealing and the GO treatment decreased to 83.1 and 79.9%, respectively. The decreases in the values of the average transmittances due to the thermal and the GO treatments were 0.1 and 3.3%, respectively. The transmittance of the Ag NWs that had been both thermally annealed and GO treated decreased by 3.9%. Because part of the GO flake was reduced due to the thermal treatment, the decrease in the average transmittance of the thermally treated, GO-treated Ag NWs was slightly larger than that of the GO-treated Ag NWs that did not undergo thermal treatment. Figure 2(b) shows the transmittances of the UV/O_3_-treated Ag NW electrodes. After the UV/O_3_ treatment, the average transmittance of the Ag NWs was 82.0%, a decrease of 1.2% in comparison with that of the pristine Ag NWs. The average transmittance of the thermally annealed Ag NWs was 82.9%, a decrease of 0.2% compared to that of the pristine Ag NWs. The degrees of decrease in the average transmittance for the thermally treated and for the UV/O_3_-treated Ag NWs were similar. However, while the average transmittance of the GO-treated Ag NWs decreased to 71.1%, that of the Ag NWs that had been both thermally annealed and GO-treated decreased to 71.3%. The average transmittances of the GO-treated Ag NWs and the thermally annealed and GO-treated Ag NWs were decreased by 8.8 and 8.0%, respectively. The differences in the average values of the transmittance were related to variations in the shapes of the Ag NWs. The degradation of the Ag NWs that had received only the UV/O_3_ treatment was interrupted by Ag debris, but the shape of Ag NWs did not change completely to that of Ag nanoparticles, resulting in a very slight decrease in the transmittance. However, the average transmittance of the GO-treated Ag NWs was significantly decreased by light scattering from the nanoparticles generated from the degradation of the Ag NWs.

**Figure 3:**
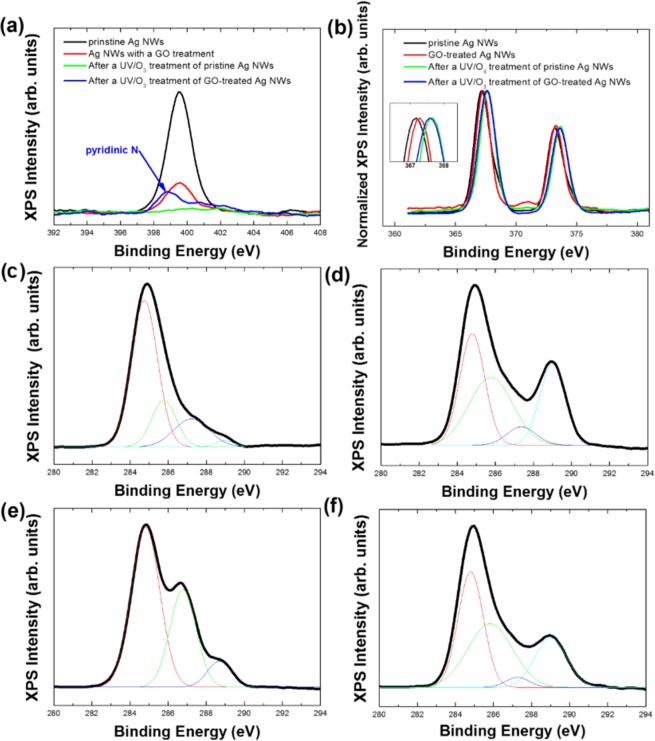
X-ray photoelectron spectroscopy (XPS) spectra of the Ag NWs with and without a GO treatment, and their XPS spectra related to the (**a**) N 1 s and the (**b**) Ag 3d orbitals after a UV/O_3_ treatment. XPS C1s spectra of several kinds of post-treated Ag NWs with and without a GO treatment, and their deconvolution peaks for (**c**) pristine Ag NWs, (**d**) Ag NWs after a UV/O_3_ treatment, (**e**) GO-covered Ag NWs, and (**f**) GO-covered Ag NWs after a UV/O_3_ treatment.

The variations in the chemical composition of the Ag NWs caused by the UV/O_3_ treatment were confirmed by using XPS spectra. Figure 3 shows the XPS spectra of elemental N, Ag, and C in the Ag NWs before and after the UV/O_3_ treatment. The intensity of the N 1 s peak for the Ag NWs was decreased after a GO treatment of the pristine Ag NWs and was almost not observed after the UV/O_3_ treatment. However, the peak related to pyridinic nitrogen in the N 1 s peak of the GO-treated Ag NWs was increased after the UV/O_3_ treatment, as shown in Fig. 3(a). The increase in the intensity of the pyridinic nitrogen peak indicates that the PVP, which contains nitrogen, was destroyed by the ozone, and that the nitrogen atoms thus generated bonded to carbon defects on the GO surface, resulting in an increase in the amount of pyridinic nitrogen. Figure 3(b) shows the variation in the Ag 3d peaks of the Ag NWs due to the GO and the UV/O_3_ treatments. The binding energy was slightly increased by 0.1 eV due to the GO treatment, and the binding energy of the Ag 3d peak for the UV/O_3_-treated Ag NWs shifted to higher energy by 0.4 eV. The increase in the binding energy indicates that the number of Ag atoms in the metal phase had increased due to the UV/O_3_ treatment and the number of Ag atoms in the oxide phase had decreased. Figure 3(c–f) show the spectra containing the C1s peaks and their decomposition spectra. The C1s spectrum of the pristine Ag NWs can be deconvoluted into four components, which correspond to C1s impurities, C-C bonds in the PVP protective layer, C-O bonds, and C=O bonds, as shown in Fig. 3(c). The intensities of the peak associated with C1s impurities and C-O bonds were decreased after the UV/O_3_ treatment, and those of the peaks associated with the C-C bonds in the PVP layer and the C=O bonds were relatively increased in comparison with those associated with C1s impurities and C-O bonds, as shown in Fig. 3(d). Figure 3(e) shows the C 1s spectrum of the GO-treated Ag NWs, which can be decomposed into three components. These three components correspond to C-C, C-O, and C=O bonds and can be attributed to the deposition of the GO flakes on the Ag NWs. After the UV/O_3_ treatment, the C1s spectrum of the GO-treated Ag NWs became similar to the C1s spectrum of the Ag NWs without GO treatment, as shown in Fig. 3(f). Therefore, while the carbon bonds existing in the GO flakes of the GO-treated Ag NWs were significantly affected by the UV/O_3_ treatment, the UV/O_3_ treatment caused huge damages on the GO surface, resulting in the dissolution of the carbon bonds existing in the GO flakes. The destruction of the GO flakes due to the UV/O_3_ treatment was confirmed by the SEM images. A number of cracks could be observed in the surfaces of the GO flakes, as shown in Fig. 5(d), which demonstrated that most of the C-C bonds of the GO flakes had been destroyed due to the UV/O_3_ treatment.

**Figure 4:**
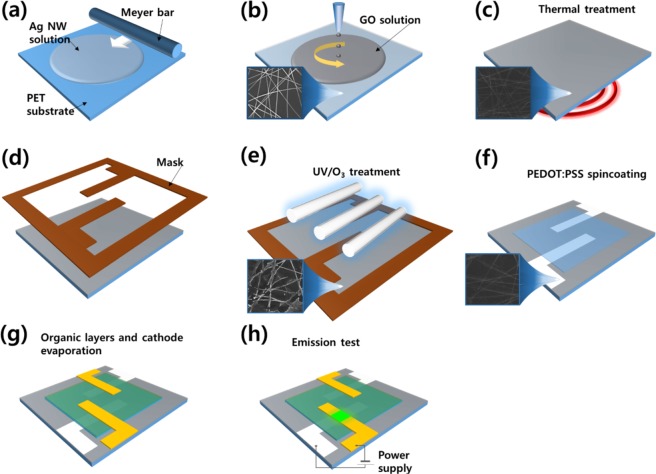
Pattern formation process of the Ag-NW electrodes by using a thermal treatment, a GO treatment, and a UV/O_3_ treatment: (**a**) bar-coating process for the Ag NWs on a PET substrate, (**b**) spin-coating process for the GO solution, (**c**) pre-heat treatment, (**d**) attachment of the UV mask, (**e**) a UV/O_3_ treatment, (**f**) spin-coating process for the PEDOT:PSS layer, (**g**) evaporation process for the organic layers and the cathode layer, and (**h**) the luminance test process for the flexible organic light-emitting device with an Ag-NW electrode.

The shapes of the Ag NWs were changed by the UV/O_3_ treatment, and their surface resistance rapidly increased with increasing UV/O_3_ treatment time. This degradation behavior of the Ag NWs due to the UV/O_3_ treatment can be further accelerated by using a thermal treatment during the initial stage. For high-density Ag NWs, their degradation can be effectively slowed by performing the GO treatment. Ag-NW-patterned electrodes can be fabricated more effectively by using the UV/O_3_ treatment than by using conventional laser patterning or photolithography. The long process time of the pattern-forming method for the Ag-NW electrodes can be sufficiently reduced by regulating the UV/O_3_ treatment conditions. Figure 4 shows the pattern formation process for a Ag-NW-patterned electrode based on the UV/O_3_ degradation of the Ag NWs and a GO treatment. The Ag-NW-patterned electrode was formed by bar-coating a Ag NW solution using a Meyer bar and then drying it in the atmosphere for 1 h. After the Ag-NW-patterned electrode had been dried, a GO flake dispersion solution was spin-coated onto the Ag-NW-patterned electrode at 2000 rpm for 30 s. After the spin coating, thermal treatment was performed at 90 °C for 10 min. The annealing process was conducted to remove residual solvents and to decrease the contact resistance of the Ag NWs. Furthermore, the annealing process during the pattern formation process weakened the passivation of the Ag NWs, resulting in an increase in the efficiency of the subsequent UV/O_3_ treatment. After the heat treatment process, a pattern mask was attached to the Ag-NW electrode; then, a UV/O_3_ treatment was performed. The parts of the Ag-NW electrode exposed to UV light deteriorated, and the parts covered by the mask did not. Then, a PEDOT:PSS layer was coated on the patterned Ag-NW electrode, and organic layers were deposited to fabricate an organic light-emitting device (OLED).

**Figure 5:**
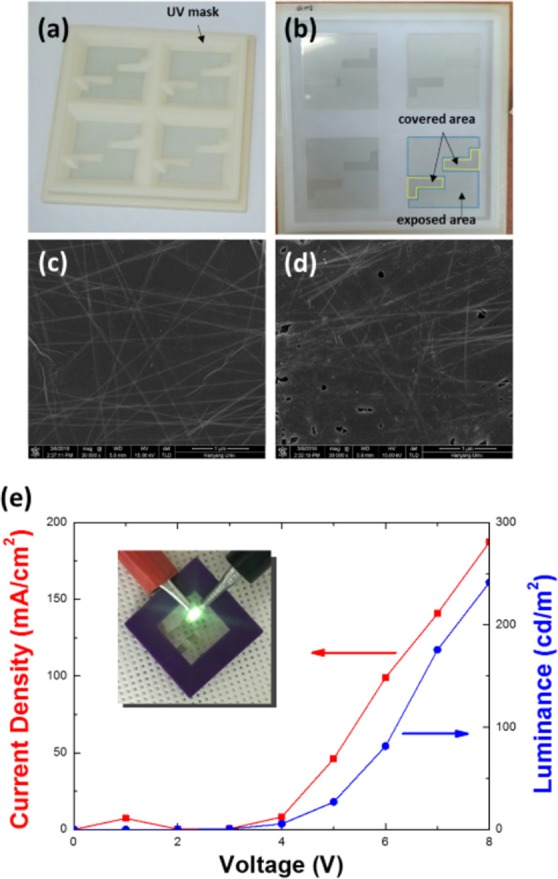
Photographs of (**a**) a mask attached on the Ag-NW electrode and (**b**) the patterns on the Ag-NW electrode due to the UV/O_3_ treatment. Scanning electron microscopy images of the (**c**) covered and the (**d**) exposed areas. (**e**) Current density-voltage and luminance-voltage characteristics of the organic light-emitting device using the patterned Ag-NW electrode. The inset shows a photograph of a flexible OLED fabricated by using an electrode with an Ag-NW pattern formed by using GO, thermal and UV/O_3_ treatments.

Figure 5(a) shows the pattern mask attached to a substrate with a Ag-NW-patterned electrode, and Fig. 5(b) presents a photograph that substrate after UV/O_3_ treatment of Ag-NW-patterned electrode. The exposed area of the Ag NW electrode was indicated by a blue dotted area, and the covered areas were denoted by yellow dotted areas. While the exposed area became blurred because of the light scattering due to the formation of the silver nanoparticles resulting from the destruction of the Ag NWs, the covered area maintained transparent. Figure 5(c,d) show SEM images of the covered and the exposed areas irradiated by UV light, respectively. The surfaces of the GO flakes on the Ag NWs were destroyed by the UV/O_3_ treatment, and the Ag NWs covered by the GO flakes were also partially destroyed, resulting in a deterioration of the Ag-NW network, as shown in Fig. 5(d). On the other hand, the parts of the Ag-NW electrode that had been covered by using the mask maintained almost the initial shapes of the Ag NWs during the UV/O_3_ treatment. While the Ag-NW electrode without the GO treatment was not completely destroyed due to the effect of Ag debris, as shown in Fig. 1(f), the degradation of the Ag NWs in the GO-treated Ag-NW electrode was not hindered by the Ag debris.

OLEDs were fabricated on the GO-treated Ag-NW-patterned electrode on the PET substrate by using the process shown in Fig. 4. Figure 5(e) shows the current-voltage and the luminance-voltage characteristics of such OLEDs. The inset in Fig. 5(e) presents a photograph of the flexible OLED with the GO-covered, Ag-NW-patterned electrode. The flexible OLED fabricated on the PET substrate was fixed to a purple plastic guide and then connected to the power supply to turn it on. Even though the surface of the Ag-NW-patterned electrode was still very rough and the luminance of the OLED was relatively low, the effectiveness of our novel method for forming Ag-NW patterns on electrodes, after pattern optimization, for use in highly efficient, flexible OLEDs has been demonstrated.

## Discussion

The process for forming Ag-NW patterns on electrodes, which used GO and UV/O_3_ treatments, and the electrical, structural, and optical properties of those electrodes were investigated. The Ag-NW electrodes were degraded by the UV/O_3_ treatment, and their sheet resistances were significantly increased. While the degradation in the high-density Ag NWs was hindered by deterioration interferences, resulting in an arduous pattern formation process, the pre-thermal and the GO treatments accelerated that degradation and reduced the deterioration interferences. SEM images of the Ag NWs that had been degraded by the UV/O_3_ treatment showed that the increased sheet resistance and the decreased transmittance were caused by morphological deformation resulting from the degradation of the Ag NWs. The GO flakes coated on the Ag NWs caused the degradation in the high-density Ag NWs to be enhanced; those flakes also aided the formation of patterns of high-density Ag NWs on an electrode. The potential applications of Ag-NW-patterned electrodes, when the degradation resulting from variations in the sheet resistance, the transmittance, and the destruction of chemical bonds caused by the UV/O_3_ treatment are controlled, were clearly identified. The performances of flexible OLEDs fabricated using Ag-NW-patterned electrodes on PET substrates, which are inexpensive, may open a wide range of applications in large-area electronic and optoelectronic devices.

## Supplementary information

Flexible, transparent patterned electrodes based on graphene oxide/silver nanowire nanocomposites fabricated utilizing an accelerated ultraviolet/ozone process to control silver nanowire degradation
