# Supplementary material for: Flexible, transparent patterned electrodes based on graphene oxide/silver nanowire nanocomposites fabricated utilizing an accelerated ultraviolet/ozone process to control silver nanowire degradation
Source: Sci Rep. 2019 Apr 2;9:5527. doi: 10.1038/s41598-019-41909-4 (PMC6445337; doi:10.1038/s41598-019-41909-4)
Supplement: Supplementary file 1 — Flexible, transparent patterned electrodes based on graphene oxide/silver nanowire nanocomposites fabricated utilizing an accelerated ultraviolet/ozone process to control silver nanowire degradation [file 41598_2019_41909_MOESM1_ESM.docx]

Flexible, transparent patterned electrodes based on graphene oxide/silver nanowire nanocomposites fabricated utilizing an accelerated ultraviolet/ozone process to control silver nanowire degradation

Dong Chul Choo^1^, Sang Kyung Bae^2^, and Tae Whan Kim^1,2^^[[1]](#footnote-1)^*

^1^Department of Electronics and Computer Engineering, Hanyang University, Seoul 04763, Republic of Korea

^2^Department of Information Display Engineering, Hanyang University, Seoul 04763, Republic of Korea

**Table S1|** Percent contributions of the C-C, the C-O, and the C=O sub-peaks to the C1s spectrum after various treatments of the Ag-NW electrode.

| Sub-peaks  Treatment type | C-C | C-O | C=O |
| --- | --- | --- | --- |
| as-grown | 86.2 | 13.8 | 0 |
| thermal treatment at 90°C for 10 min | 83.5 | 16.5 | 0 |
| thermal treatment at 110°C for 10 min | 85.1 | 12.6 | 2.3 |
| UV/O₃ treatment for 2 h | 80.9 | 8.2 | 10.9 |
| thermal treatment at 90°C for 10 min and UV/O₃ treatment for 2 h | 76.8 | 8.1 | 15.0 |
| thermal treatment at 90°C for 10 min and UV/O₃ treatment for 4 h | 75.6 | 7.0 | 17.4 |

**Figure S1** | X-ray photoelectron spectroscopy (XPS) spectra related to the C 1s orbitals of the Ag NWs with and without the pre-thermal and the UV/O_3_ treatment and their decomposed peaks: (a) pristine Ag NWs, (b) Ag NWs after the pre-thermal treatment at 90ºC for 10 min, (c) Ag NWs after the pre-thermal treatment at 110^º^C for 10 min, (d) Ag NWs after the UV/O_3_ treatment for 2 h, (e) Ag NWs after the pre-thermal treatment at 90ºC for 10 min and the UV/O_3_ treatment for 2 h, and (f) Ag NWs after the pre-thermal treatment at 90ºC for 10 min and the UV/O_3_ treatment for 4 h.


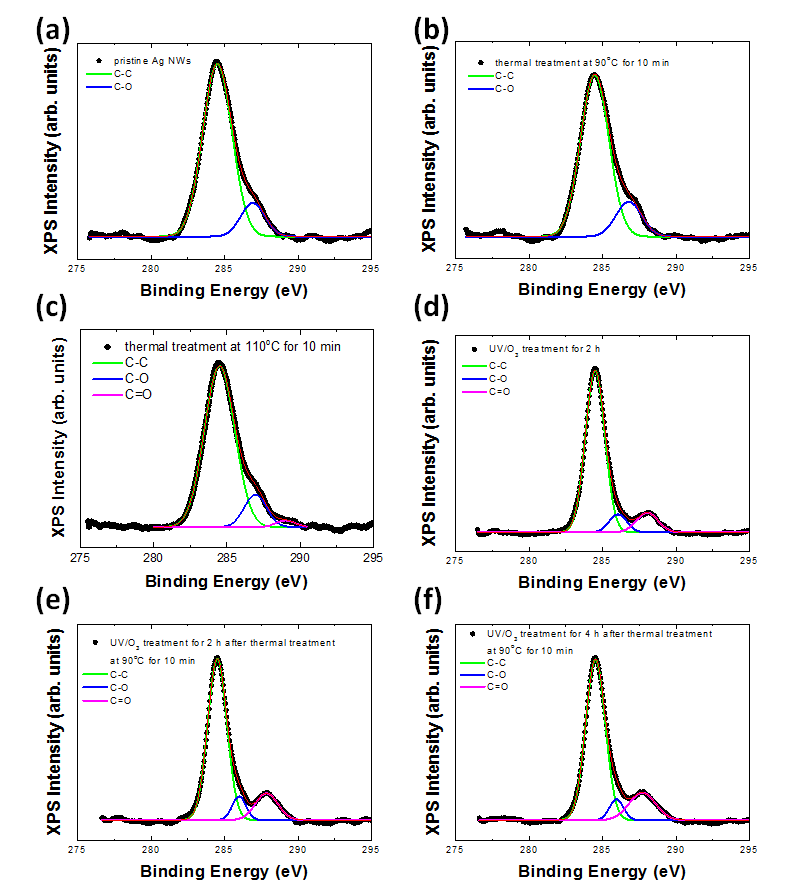


1. *Corresponding author e-mail: twk@hanyang.ac.kr [↑](#footnote-ref-1)
